# Supplementary material for: Preparatory attentional templates in prefrontal and sensory cortex encode target-associated information
Source: eLife. 2025 Sep 8;14:RP104041. doi: 10.7554/eLife.104041 (PMC12416899; doi:10.7554/eLife.104041)
Supplement: Supplementary file 1. [file elife-104041-supp1.docx]

**Supplementary File 1**

| Whole-brain searchlight results of brain regions showing significant decoding during the ***search cue*** period. | | | | |  |
| --- | --- | --- | --- | --- | --- |
|  |  |  |  |  |  |
| Brain region | L/R | Cluster size (voxel number) | Peak MNI coordinate (x,y,z) | Z-score |  |
|  |  |  |  |  |  |
| ***Decoding of face information*** |  |  |  |  |  |
| Fusiform gyrus | R | 616 | 42 -48 -8 | 4.74 |  |
| Middle frontal gyrus | L | 2039 | -22 32 30 | 4.21 |  |
| Middle frontal gyrus | R | 1170 | 28 30 34 | 3.86 |  |
| Inferior parietal sulcus | R | 690 | 38 -48 52 | 3.82 |  |
| Superior parietal lobule | R |  | 30 -50 54 | 3.72 |  |
| Middle temporal gyrus | R | 220 | 42 -46 18 | 3.26 |  |
| Middle occipital gyrus | L | 182 | -14 -96 -6 | 3.67 |  |
| Calcarine | L |  | -20 -94 2 | 3.31 |  |
| Calcarine | R | 182 | 20 -92 2 | 3.39 |  |
| Inferior occipital gyrus | R |  | 32 -78 -8 | 3.38 |  |
| Precuneus | L | 424 | -10 -52 38 | 3.58 |  |
|  |  |  |  |  |  |
| ***Decoding of scene information*** |  |  |  |  |  |
| Insula | R | 385 | 38 12 0 | 3.91 |  |
| Inferior frontal gyrus | R |  | 36 24 14 | 3.66 |  |
| Inferior frontal gyrus | L | 268 | -38 30 28 | 3.50 |  |
| Middle frontal gyrus | L |  | -34 34 16 | 3.44 |  |
| Peak voxel coordinate is defined in MNI152 standard space. Voxel size: 2.0 2.0 2.0 mm mm mm; L, left; R, right; MNI, Montreal Neurological Institute. | | | | |  |
|  |  |  |  |  |  |
