# Supplementary material for: Preparatory attentional templates in prefrontal and sensory cortex encode target-associated information
Source: eLife. 2025 Sep 8;14:RP104041. doi: 10.7554/eLife.104041 (PMC12416899; doi:10.7554/eLife.104041)
Supplement: Supplementary file 2. [file elife-104041-supp2.docx]

**Supplementary File 2**

| Whole-brain searchlight results of brain regions showing significant decoding during the ***search*** ***delay*** period. | | | | |  |
| --- | --- | --- | --- | --- | --- |
|  |  |  |  |  |  |
| Brain region | L/R | Cluster size (voxel number) | Peak MNI coordinate (x,y,z) | Z-score |  |
|  |  |  |  |  |  |
| ***Decoding of face information*** |  |  |  |  |  |
| None |  |  |  |  |  |
|  |  |  |  |  |  |
| ***Decoding of scene information*** |  |  |  |  |  |
| Retrosplenial cortex | L | 3065 | -14 -54 12 | 4.73 |  |
| Retrosplenial cortex | R | 914 | 14 -48 12 | 3.98 |  |
| Precentral | R | 491 | 50 2 42 | 3.75 |  |
| Inferior frontal gyrus | R |  | 48 6 32 | 3.54 |  |
| Precentral | L | 192 | -42 -2 32 | 2.83 |  |
| Precentral | L | 54 | -36 -22 54 | 2.20 |  |
| Superior frontal gyrus | R | 605 | 18 36 38 | 3.08 |  |
| Precuneus | R | 205 | 10 -60 46 | 2.45 |  |
| Parahippocampal | L | 83 | -32 -36 -16 | 2.34 |  |
| Parahippocampal | R | 165 | 28 -58 -12 | 2.17 |  |
| Peak voxel coordinate is defined in MNI152 standard space. Voxel size: 2.0 2.0 2.0 mm mm mm; L, left; R, right; MNI, Montreal Neurological Institute. | | | | |  |
|  |  |  |  |  |  |
