# Supplementary material for: Preparatory attentional templates in prefrontal and sensory cortex encode target-associated information
Source: eLife. 2025 Sep 8;14:RP104041. doi: 10.7554/eLife.104041 (PMC12416899; doi:10.7554/eLife.104041)
Supplement: Supplementary file 3. [file elife-104041-supp3.docx]

**Supplementary File 3**

| Univariate contrasts related to scene-validity during the search period. | | | | |  |
| --- | --- | --- | --- | --- | --- |
|  |  |  |  |  |  |
| Brain region | L/R | Cluster size (voxel number) | Peak MNI coordinate (x,y,z) | Z-score |  |
|  |  |  |  |  |  |
| ***Contrast: scene-invalid vs. scene-valid*** | | | | |  |
| Inferior frontal gyrus | L | 832 | -40 8 28 | 4.34 |  |
| Inferior frontal gyrus | R | 1075 | 42 8 28 | 3.70 |  |
| Inferior parietal sulcus | L | 318 | -30 -64 40 | 3.68 |  |
| Inferior parietal sulcus | R | 561 | 36 -62 34 | 3.27 |  |
| Insula | L | 88 | -28 26 -4 | 3.36 |  |
| Insula | R | 296 | 30 32 -8 | 3.53 |  |
| Anterior cingulate cortex (ACC) | R | 582 | 6 40 40 | 3.04 |  |
|  |  |  |  |  |  |
| ***Contrast: scene-valid vs. scene-invalid*** | | | | |  |
| Superior parietal lobule | L | 1512 | -24 -44 74 | 3.93 |  |
| Supramarginal | L | 2980 | -62 -34 32 | 3.61 |  |
| Superior parietal lobule | R | 6044 | 26 -40 54 | 3.69 |  |
| Supramarginal | R |  | 64 -36 42 | 3.47 |  |
| Middle occipital gyrus | L | 1465 | -38 -86 6 | 3.19 |  |
| Inferior occipital gyrus | R | 1517 | 42 -86 -6 | 3.10 |  |
| Peak voxel coordinate is defined in MNI152 standard space. Voxel size: 2.0 2.0 2.0 mm mm mm; L, left; R, right; MNI, Montreal Neurological Institute. | | | | |  |
|  |  |  |  |  |  |
