# Supplementary material for: Preparatory attentional templates in prefrontal and sensory cortex encode target-associated information
Source: eLife. 2025 Sep 8;14:RP104041. doi: 10.7554/eLife.104041 (PMC12416899; doi:10.7554/eLife.104041)
Supplement: Supplementary file 4. [file elife-104041-supp4.docx]

**Supplementary File 4**

| Mean (SE) number of voxels in each ROI. | | | |
| --- | --- | --- | --- |
| ROI | | Left hemisphere | Right hemisphere |
| IFJ | PEF | 36 (5) | 47 (7) |
|  | IFJp | 29 (4) | 26 (4) |
|  | IFJa | 34 (4) | 38 (5) |
| Frontal | FEF | 83 (5) | 86 (5) |
|  | vLPFC | 269 (11) | 180 (9) |
|  | dLPFC | 44 (4) | 210 (11) |
| Parietal | SPL | 202 (7) | 205 (10) |
|  | IPS | 157 (6) | 106 (6) |
|  | IPL | 119 (5) | 155 (8) |
| Visual | FFA | 65 (6) | 113 (9) |
|  | PPA | 126 (10) | 163 (11) |
|  | V1 | 122 (7) | 175 (7) |
